# A solvable model for symmetry-breaking phase transitions

**DOI:** 10.1038/s41598-023-40704-6

**Published:** 2023-08-23

**Authors:** Shatrughna Kumar, Pengfei Li, Liangwei Zeng, Jingsong He, Boris A. Malomed

**Affiliations:** 1 Department of Physical Electronics, School of Electrical Engineering, Faculty of Engineering, and Center for Light-Matter Interaction, Tel Aviv University, Tel Aviv, 69978 Israel; 2 Department of Physics, Taiyuan Normal University, Jinzhong, 030619 China; 3 Department of Basic Course, Guangzhou Maritime University, Guangzhou, 510725 China; 4 Institute for Advanced Study, Shenzhen University, Shenzhen, Guangdong China; 5 Instituto de Alta Investigación, Universidad de Tarapacá, Casilla 7D, Arica, Chile

**Keywords:** Nonlinear phenomena, Nonlinear optics, Bose-Einstein condensates

## Abstract

Analytically solvable models are benchmarks in studies of phase transitions and pattern-forming bifurcations. Such models are known for phase transitions of the second kind in uniform media, but not for localized states (solitons), as integrable equations which produce solitons do not admit intrinsic transitions in them. We introduce a solvable model for symmetry-breaking phase transitions of both the first and second kinds (alias sub- and supercritical bifurcations) for solitons pinned to a combined linear-nonlinear double-well potential, represented by a symmetric pair of delta-functions. Both self-focusing and defocusing signs of the nonlinearity are considered. In the former case, exact solutions are produced for symmetric and asymmetric solitons. The solutions explicitly demonstrate a switch between the symmetry-breaking transitions of the first and second kinds (i.e., sub- and supercritical bifurcations, respectively). In the self-defocusing model, the solution demonstrates the transition of the second kind which breaks antisymmetry of the first excited state.

## Introduction

### The topic: spontaneous symmetry breaking in nonlinear systems

Dynamics of collective excitations in physical systems is determined by the interplay of the underlying diffraction or dispersion, nonlinear self-interactions of the fields or wave functions, and potentials acting on the fields. In this context, it is commonly known that the ground state (GS) of linear systems reproduces the symmetry of the underlying potential, while excited states may realize other representations of the same symmetry^1^. In particular, the wave function of a particle trapped in a symmetric double-well potential (DWP) is even, while the first excited state is odd.

While these basic properties are demonstrated by the linear Schrödinger equation, the dynamics of Bose-Einstein condensates (BECs) is governed, in mean-field approximation, by the Gross-Pitaevskii equation (GPE), which takes into regard interactions between particles, adding the cubic term to the Schrödinger equation for the single-particle wave function^2,3^. The repulsive or attractive interactions are represented by the cubic term with the self-defocusing (SDF) or self-focusing (SF) sign. Essentially the same model is the celebrated nonlinear Schrödinger equation (NLSE), which governs the propagation of optical waves in nonlinear media^4^ and finds a plenty of other realizations, as the universal model to govern the interplay of the weak diffraction or dispersion and cubic SF nonlinearity^5^. In optics, a counterpart of the trapping potential is the term in the NLSE which accounts for the waveguiding structure induced by a transverse profile of the refractive index.

The GS structure in models combining the DWP and SF nonlinearity follows the symmetry of the underlying potential only in the weakly nonlinear regime. A generic effect which occurs with the increase of the SF nonlinearity strength is the symmetry-breaking phase transition, which makes the GS asymmetric with respect to two wells of the DWP^6^. This effect of the spontaneous symmetry breaking (SSB) implies, *inter alia*, that the commonly known principle of quantum mechanics, according to which GS cannot be degenerate^1^, is no longer valid in the nonlinear models: obviously, the SSB gives rise to a degenerate pair of two mutually symmetric GSs, with the maximum of the wave function pinned to the left or right potential well of the underlying DWP. The same system admits a symmetric state coexisting with the asymmetric ones, but, above the SSB point, it does not represents the GS, being unstable against symmetry-breaking perturbations.

In systems with the SDF sign of the nonlinearity, the GS remains symmetric and stable, while the SSB transition breaks the *antisymmetry* of the first excited state (it is a spatially odd one, with precisely one zero of the wave function, located at the central point). The resulting state with the spontaneously broken antisymmetry keeps the zero point, which is shifted from the center to right or left.

The concept of the SSB in systems of the NLSE type with the SF nonlinearity was first proposed, in an abstract mathematical form, by Davies in 1979^7^. Another early realization of this concept was introduced in 1985 by Eilbeck, Lomdahl, and Scott, in the form of the “self-trapping model”^8^. The latter work had actually initiated systematic studies of SSB phase transitions.

In optics, the SSB was observed experimentally in a photorefractive crystal with saturable SF nonlinearity and an effective DWP waveguiding structure^9^. SSB was also predicted for photonic modes supported by a symmetrically designed plasmonic metamaterial^10^. For the self-attractive BEC loaded into a DWP trap, the symmetry-breaking transition was elaborated in Refs.^11,12^. In that context, tunnel-coupling oscillations between condensates trapped in two potential wells separated by a barrier represent the bosonic Josephson effect^13^. Experimentally, the self-trapping of a stationary state with spontaneously broken antisymmetry in a self-repulsive condensate loaded in DWP, as well as Josephson oscillations in that setup, were reported in Ref.^14^.

A ramification of the topic is SSB in dual-core systems, such as twin-core optical fibers, with the SF cubic nonlinearity acting in each core^15^. In such systems, the interplay of the SF and linear coupling between parallel cores gives rise to the SSB transition from the symmetric state to a spontaneously established one with unequal powers carried by the two cores. This type of the symmetry-breaking phase transition was studied in detail theoretically^16–21^ and recently demonstrated experimentally in a twin-core fiber^22^. In terms of the respective system of linearly-coupled NLSEs, the SSB transition is represented by the bifurcation which links symmetric and asymmetric solutions^23^. Depending on the type of the intra-core nonlinearity and the wave form under the consideration (delocalized or self-trapped), the symmetry-breaking bifurcation may be of the supercritical (alias forward) or subcritical (backward) type. The corresponding bifurcations give rise to the destabilization of the symmetric state and creation of a pair of asymmetric ones at the SSB point, which go forward or backward as stable or unstable branches, respectively, following the variation of the SSB-driving nonlinearity strength. In the latter (subcritical) case, the unstable *lower* branches of the asymmetric states normally reverse into the stable forward-going *upper* ones at certain turning points (see, e.g., Fig. 7 below). As a result, stable asymmetric states emerge subcritically, at a value of the nonlinearity strength which is smaller than that at the SSB point. Accordingly, the system is bistable in the interval between the turning and SSB points, where the symmetric and upper asymmetric states coexist as stable ones. In terms of statistical physics, the super- and subcritical bifurcations are identified as symmetry-breaking phase transitions of the second and first kinds, respectively. In the latter case, the bistability corresponds to the hysteresis between the GS and overcooled or overheated phases.

Other varieties of optical SSB effects occur in dual-core laser setups combining the SF nonlinearity with gain and loss. The theoretical model of such setups is based on a pair of linearly coupled complex Ginzburg-Landau equations with the cubic-quintic nonlinearity^24^. A spontaneously established asymmetric regime of the operation of a symmetric pair of coupled lasers was observed in Ref.^25^.

The SSB phenomenology was also predicted in models with symmetric * nonlinear potentials*, induced by spatial modulation of the local SF or SDF coefficient^26,27^. In optical waveguides, the modulation can be imposed by spatially inhomogeneous distributions of a resonant dopant, which gives rise to strong local nonlinearity^28^. In experiments with BEC, the Feshbach resonance (FR) controlled by spatially nonuniform laser illumination of the condensate may be employed to build an effective nonlinearity landscape^29–31^. Other techniques available to the experimental work with BEC make it possible to “paint” a necessary FR-induced nonlinear potential by a fast moving laser beam^32^ or a spatial light modulator^33–35^.

### The model

The use of the nonlinear potential suggests a possibility to design experimentally feasible *solvable* SSB settings, which admit exact analytical solutions for symmetric, antisymmetric, and asymmetric states. The key component of solvable models is the nonlinear term in the NLSE with coordinate *x*, which is concentrated at $x=0$, being represented by the $\delta$-function:

1 $$\begin{aligned} i\frac{\partial \psi }{\partial z}=-\frac{1}{2}\frac{\partial ^{2}\psi }{ \partial x^{2}}-\delta \left( x\right) \left( \varepsilon +\sigma |\psi |^{2}\right) \psi . \end{aligned}$$

This model is formulated in terms of optics, with the evolution along propagation distance *z* under the action of the real nonlinearity coefficient $\sigma$, scaled to be $\sigma =+1$ or $-1$, which corresponds, respectively, to the SF or SDF sign of the nonlinearity. In that case, the $\delta$-function term represents a narrow layer of an optical material with strong cubic susceptibility (e.g., AlGaAs, whose susceptibility exceeds that of silica by a factor $\simeq 700$^36^) embedded in the linear planar waveguide, provided that the width of the layer is small in comparison with that of self-trapped light beams propagating in the waveguide. This setting can be readily implemented in the experiment, as the typical width of spatial solitons is measured in tens of microns^37^. In that case, the linear trapping potential, $-\varepsilon \delta (x)$, present in Eq. (1), is relevant too, as the linear refractive index of materials such as AlGaAs is much higher than the background value in the host material (silica). As concerns the sign of the nonlinearity, the consideration of the SDF layer is also interesting, as semiconductor materials may demonstrate negative nonlinear susceptibility.

The same Eq. (1), with *z* replaced by time *t*, applies to BEC, with the $\delta$-function potential induced by the FR-inducing laser beam tightly focused at $x=0$. The same optical beam also induces the linear potential represented by coefficient $\varepsilon$. In a similar context, Eq. (1) with $\varepsilon =0$ was first introduced, as a model of a nonlinear bosonic junction, in Ref.^38^. Further, a model of the matter-wave soliton interferometer with a nonlinear soliton splitter corresponds to $\varepsilon <0$ and $\sigma =-1$ in Eq. (1)^39^.

Equation (1) gives rise to the exact solution for a family of self-trapped states (solitons) pinned to the delta-functional potential:

2 $$\begin{aligned} \psi _{0}(x,z)=U_{0}(x)\exp (ikz), \end{aligned}$$

where *k* is an arbitrary propagation constant, and the shape function is

3 $$\begin{aligned} U_{0}(x)=\sqrt{\sigma \left( \sqrt{2k}-\varepsilon \right) }\exp \left( - \sqrt{2k}|x|\right) . \end{aligned}$$

The self-trapped modes are characterized by their integral power,

4 $$\begin{aligned} P_{0}=\int _{-\infty }^{+\infty }U_{0}^{2}(x)dx=\sigma \left( 1-\frac{ \varepsilon }{\sqrt{2k}}\right) , \end{aligned}$$

which is a dynamical invariant of Eq. (1).

The well-known Vakhitov-Kolokolov (VK) criterion, $dP/dk>0$^40–43^, immediately implies that the family of solutions (3) in the case of the SF nonlinearity, $\sigma =+1$, and $\varepsilon >0$ is stable in its entire existence region, $k>\varepsilon ^{2}/2$ (and completely unstable if the linear potential is repulsive, with $\varepsilon <0$). For localized states supported by the SDF nonlinearity, with $\sigma =-1$, the VK stability criterion is replaced by the anti-VK one^44^, $dP/dk<0$. Accordingly, in this case the localized states (3) are also stable in their entire existence region, which is $0<k<\varepsilon ^{2}/2$. The definition of the power given by Eq. (4) demonstrates that the bound states pinned to $\delta$-function potential with the SF sign of the nonlinearity exist in the interval of $0<P_{0}<1$, while the competition of the linear attractive potential and SDF nonlinear term gives rise to the bound states in the entire range of $0<P_{0}<\infty$.

An exceptional case is the one corresponding to $\sigma =+1$ (SF) and $\varepsilon =0$ (no linear potential), for which Eq. (4) demonstrates the degeneracy of the localized states, whose power takes the single value, $P_{0}=1$, which does not depend on *k*. This property implies that the corresponding family represents a specific example of Townes solitons (a commonly known family of Townes solitons is one produced by localized solutions of two-dimensional NLSE with the spatially uniform cubic SF nonlinearity^45^). Because Townes solitons, with their single value of the power, have $dP/dk=0$, the VK criterion predicts that they correspond to a border between the stability and instability. It is known that, in fact, the Townes solitons are subject to the subexponentially commencing instability, which eventually leads to the onset of the critical collapse (emergence of a local singularity after a finite propagation distance)^42,43^.

It is also worthy to mention the value of the Hamiltonian of the pinned state (3),

5 $$\begin{aligned} H_{0}= & {} \frac{1}{2}\int _{-\infty }^{+\infty }\left| \frac{\partial \psi _{0}}{\partial x}\right| ^{2}dx-\left[ \varepsilon \left| \psi _{0}\left( x=0\right) \right| ^{2}+\frac{\sigma }{2}\left| \psi _{0}\left( x=0\right) \right| ^{4}\right] \nonumber \\= & {} -\frac{\sigma \varepsilon }{2}\left( \sqrt{2k}-\varepsilon \right) \end{aligned}$$

[the Hamiltonian is another dynamical invariant of Eq. (1)]. Note that the existence condition for solution (3), $\sigma \left( \sqrt{2k}-\varepsilon \right) >0$, implies $H_{0}<0$ for $\varepsilon >0$, hence the localized solution represents a true bound state with the negative energy.

The possibility to produce exact analytical solutions for localized states pinned to the $\delta$-function nonlinear potential suggests a possibility to design a solvable DWP model based on a set of two $\delta$-functions, separated by distance which may be set equal to 1 by means of rescaling:

6 $$\begin{aligned} i\frac{\partial \psi }{\partial z}=-\frac{1}{2}\frac{\partial ^{2}\psi }{ \partial x^{2}}-\left[ \delta \left( x+\frac{1}{2}\right) +\delta \left( x- \frac{1}{2}\right) \right] \left( \varepsilon +\sigma |\psi |^{2}\right) \psi . \end{aligned}$$

The equation for stationary states is produced by the substitution of expression (2) in Eq. (6):

7 $$\begin{aligned} kU-\frac{1}{2}\frac{d^{2}U}{dx^{2}}-\left[ \delta \left( x+\frac{1}{2} \right) +\delta \left( x-\frac{1}{2}\right) \right] \left( \varepsilon +\sigma U^{2}\right) U=0. \end{aligned}$$

The Hamiltonian corresponding to Eq. (6) is

8 $$\begin{aligned} H=\frac{1}{2}\int _{-\infty }^{+\infty }\left| \frac{\partial \psi }{ \partial x}\right| ^{2}dx-\sum _{\pm }\left[ \varepsilon \left| \psi \left( x=\pm \frac{1}{2}\right) \right| ^{2}+\frac{\sigma }{2}\left| \psi \left( x=\pm \frac{1}{2}\right) \right| ^{4}\right] , \end{aligned}$$

cf. Eq. (5). The physical implementation of the model in optics and BEC is straightforward: in the former case, one can embed two parallel nonlinear layers in the linear waveguide, while in the former case the necessary configuration may be created by two tightly focused FR-inducing laser beams.

A particular case of Eq. (6) with $\varepsilon =0$ was introduced, in the context of BEC, in Ref.^46^. Exact solutions for symmetric, antisymmetric, and, which is most interesting, asymmetric stationary wave functions were produced in that work, demonstrating a very peculiar feature, namely, an SSB bifurcation of the *extreme subcritical type*, in which backward-going branches of unstable states never turn forward and, accordingly, never become stable. In other words, it is a unique example of the symmetry-breaking phase transition of the first kind which does not produce any stable phase past the transition point, and gives rise to a fully unstable overcooled phase, represented by the completely unstable asymmetric states.

Recently, another example of such an anomalous phase transition was found in Ref.^47^ in the study of dual-core couplers with the SF nonlinearity and fractional diffraction, represented by operator $\left( -\partial ^{2}/\partial x^{2}\right) ^{\alpha /2}$, with *Lévy index* $\alpha$^48^, acting in each core. In that case, the extreme subcritical SSB bifurcation takes place at $\alpha =1$, which is the border between the normal symmetry-breaking phase transition of the first kind at $1<\alpha <2$, and full instability of the system, driven by the supercritical collapse, at $\alpha <1$. However, the fractional-coupler model cannot be solved analytically, on the contrary to Eq. (7).

### Objectives of the work

Our purpose is to produce an analytical solution of the full model, with the combined linear-nonlinear $\delta$-functional DWP in Eq. (6). The linear terms are represented by $\varepsilon >0$ (the attractive potential), while both SF and SDF signs of the nonlinearity, $\sigma =\pm 1$, will be addressed. For $\sigma =+1$, the solution explicitly demonstrates gradual switch from the extreme subcritical bifurcation to the supercritical one via a regular subcritical bifurcation, in which the backward-going (lower) branches of unstable asymmetric states reverse into stable upper branches at turning points. For $\sigma =-1$ the results are more straightforward, corroborating the stability of the symmetric GS and the occurrence of the supercritical antisymmetry-breaking transition in the first excited state.

While the asset of the model is its analytical solvability, some results are produced in a numerical form, using Eqs. (6) and (7) with a regularized $\delta$-function,

9 $$\begin{aligned} {\tilde{\delta }}(x)=\left( \pi w\right) ^{-1/2}\exp \left( -x^{2}/w^{2}\right) , \end{aligned}$$

defined by a small width *w* (in most cases, $w=0.01$ was used, which is 1/100 of the distance between the two $\delta$-funtions). In this connection, it is relevant to mention that the realization of the present model as the optical waveguide implies that a characteristic value of the separation between the two narrow attractive layers may be $\sim 50$ ${\mathrm {\mu }}$m, hence $w=0.01$ corresponds to the layer’s thickness $\sim 0.5$ ${\mathrm {\mu }}$m. In view of the above-mentioned possibility to use a material with the nonlinear susceptibility exceeding that in the bulk waveguide by a factor $\sim 700$, this thickness will be sufficient to provide the requires nonlinearity. In the case of the realization in BEC, a relevant size of the separation may be $\sim 10$ ${\mathrm {\mu }}$m. Then, the nearly-delta-functional potential may be induced by a laser beam focused on a spot of size $\sim$ 0.5 ${\mathrm {\mu }}$m, which will correspond to $w\simeq 0.05$, in the scaled units.

The comparison with the numerical solutions is relevant to check how well the solvable model represents a realistic one, with the finite width *w* of the potential wells, and also to test predictions for stability of the symmetric, antisymmetric, and asymmetric solitons pinned to the $\delta$ -functional DWP. The analytical and numerical results are summarized in the next section, and are discussed in the concluding one.

## Results

### Exact analytical solutions for the symmetric, asymmetric, and antisymmetric states

#### The self-focusing nonlinearity

The fact that Eq. (7) is linear at $x\ne \pm 1/2$ makes it possible to construct obvious solutions in these areas, as $\exp \left( - \sqrt{2k}\left| x+1/2\right| \right)$ and $\exp \left( -\sqrt{2k} \left| x-1/2\right| \right)$ at $x<-1/2$ and $x>+1/2$, respectively, and a combination of these terms at $|x|<1/2$. At points $x=\pm 1/2$, the solutions are matched by the continuity condition for *U*(*x*) and the jump condition for the derivative *dU*/*dx*,

10 $$\begin{aligned} dU/dx|_{x\pm 1/2=+0}-dU/dx|_{x\pm 1/2=-0}=-2\left( \varepsilon +\sigma U^{2}\right) U|_{x\pm 1/2=0}. \end{aligned}$$

The generic solution satisfying these conditions can be looked for as

11 $$\begin{aligned} U(x)= & {} U_{1}(k)\exp \left( \sqrt{2k}\left( x+\frac{1}{2}\right) \right) ,\, {\textrm{at}}\,x<-\frac{1}{2}, \end{aligned}$$

12 $$\begin{aligned} U(x)= & {} U_{2}(k)\exp \left( \sqrt{2k}\left( -\left| x-\frac{1}{2} \right| \right) \right) ,\,{\textrm{at}}\,x>\frac{1}{2}, \end{aligned}$$

13 $$\begin{aligned} U(x)= & {} U_{2}(k)\frac{\sinh \left( \sqrt{2k}\left( x+1/2\right) \right) }{ \sinh \left( \sqrt{2k}\right) }+U_{1}(k)\frac{\sinh \left( \sqrt{2k}\left( 1/2-x\right) \right) }{\sinh \left( \sqrt{2k}\right) },{\textrm{at}}\,|x|<\frac{ 1}{2}, \end{aligned}$$

where amplitudes $U_{1}(k)$ and $U_{2}(k)$ should be found from the substitution of ansatz (11)–(13) in Eq. (10). For this stationary solution, the value of Hamiltonian (8) is

14 $$\begin{aligned} H=2kP-\varepsilon \left( U_{1}^{2}+U_{2}^{2}\right) -\frac{\sigma }{2}\left( U_{1}^{4}+U_{2}^{4}\right) , \end{aligned}$$

where *P* is the integral power, defined as per Eq. (4).

First, it is easy to find the exact solutions for symmetric states in the SF model ($\sigma =+1$), with equal amplitudes $U_{1}(k)=U_{2}(k)\equiv U_{ {\textrm{symm}}}(k)$:

15 $$\begin{aligned} U_{{\textrm{symm}}}(k)=\sqrt{\sigma \left[ E(\varepsilon ,k)-1\right] /S(k)}, \end{aligned}$$

where

16 $$\begin{aligned} S(k)\equiv & {} \sqrt{\frac{2}{k}}\sinh \left( \sqrt{2k}\right) , \end{aligned}$$

17 $$\begin{aligned} E(\varepsilon ,k)\equiv & {} \exp \left( \sqrt{2k}\right) -\varepsilon \sqrt{ \frac{2}{k}}\sinh \left( \sqrt{2k}\right) . \end{aligned}$$

**Figure 1:**
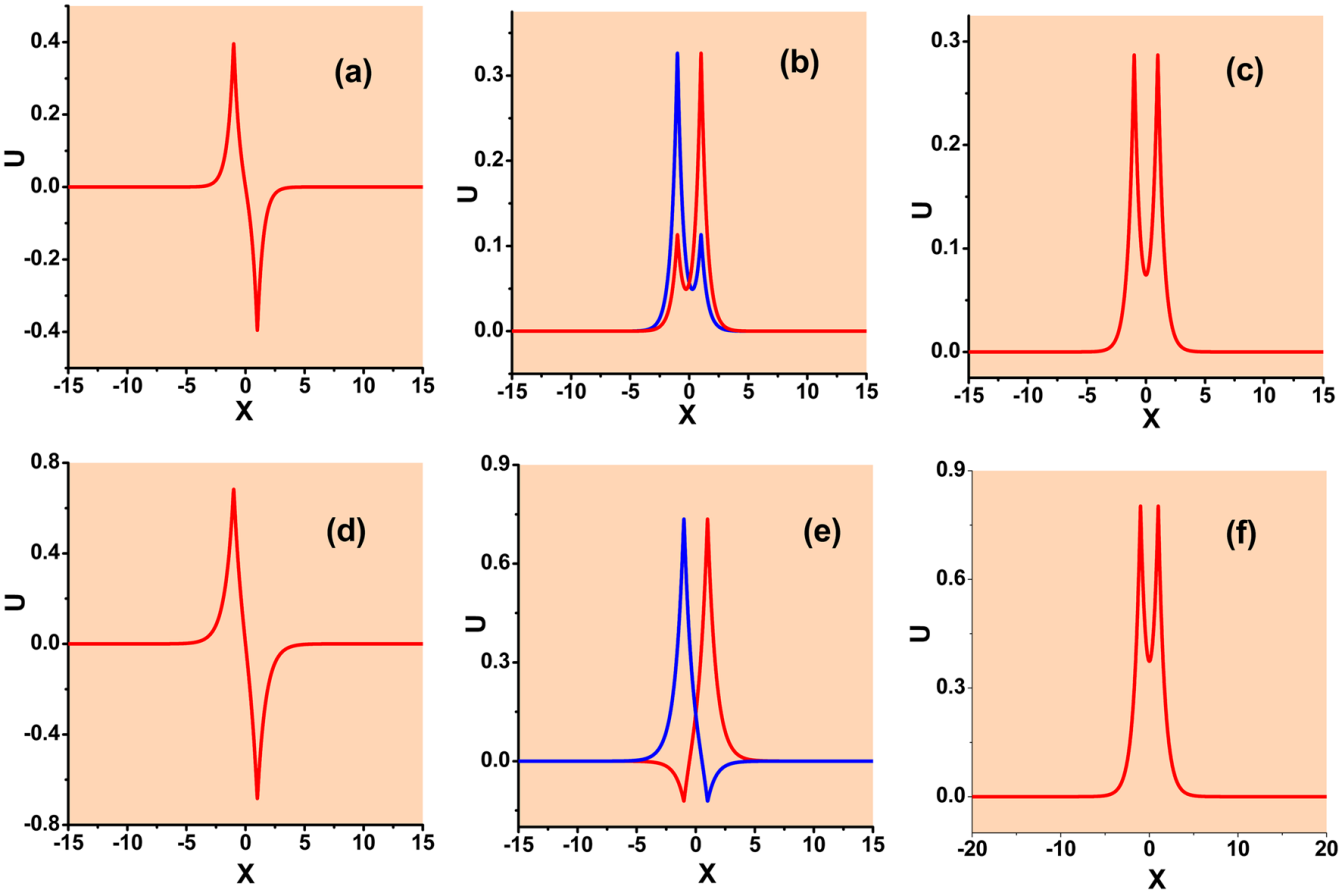
(**a**–**c**) Typical examples of antisymmetric, asymmetric, and symmeric bound states (solitons) produced by the numerical solution of Eq. (7) with the $\delta$-functions approximated by expression (9), the SF nonlinearity ($\sigma =+1$), and parameters $\varepsilon =2$, $k=2.1$. In panel (**b**) two asymmetric states are plotted, which are mirror images of each other. The antisymmetric and symmetric states are unstable, while the asymmetric one is stable. (**d**–**f**) Typical examples of antisymmetric, broken-antisymmetry, and symmetric bound states for the SDF nonlinearity ($\sigma =-1$) and $\varepsilon =2$, $k=1$. In panel (**e**), two states with broken antisymmetry are mirror images of each other. The antisymmetric state is unstable, while the ones with broken antisymmetry and unbroken symmetry are stable.

A typical example of a symmetric bound state (soliton), for $\varepsilon =2$ and $k=2.1$, is displayed in Fig. 1c. This plots is produced by the numerical solution of Eq. (7), being virtually indistinguishable from its counterpart given by the analytical solution, as provided by Eqs. (11)–(13) and (15).

Because *S*, as defined by Eq. (16), is always positive, the solution given by Eq. (15) for $\sigma =+1$ and $-1$ exists for $E(\varepsilon ,k)>1$ and $E(\varepsilon ,k)<1$, respectively. As it follows from Eq. (17), this condition implies that, in the case of SF nonlinearity, the symmetric state with given propagation constant *k* exists if the strength of the linear $\delta$-function potential does not exceed a maximum value,

18 $$\begin{aligned} \varepsilon <\left( \varepsilon _{\max }\right) _{{\textrm{symm}}}\equiv \frac{ \sqrt{2k}}{1+\exp \left( -\sqrt{2k}\right) }. \end{aligned}$$

**Figure 2:**
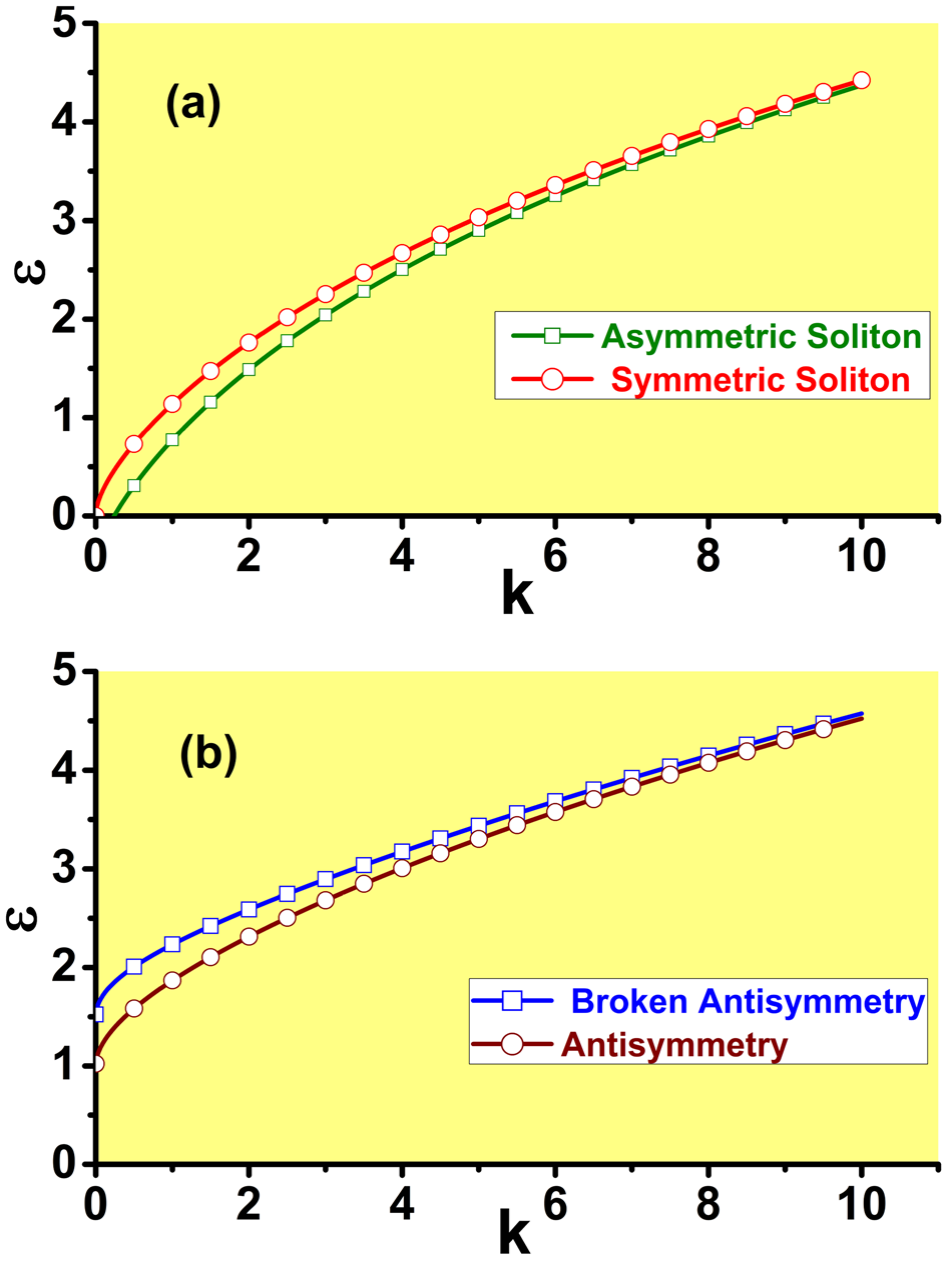
(**a**) In the model with the SF nonlinearity, $\sigma =+1$, the symmetric bound states with amplitudes (15) exist beneath the boundary in the plane of $\left( k,\varepsilon \right)$ displayed by the red curve, which is produced by Eq. (18). The asymmetric states, with the amplitudes given by Eqs. (22) and (23), exist beneath the green boundary, which is produced by Eq. (24). (**b**) In the model with the SDF nonlinearity, $\sigma =-1$, the antisymmetric bound states with amplitudes (15) exist above the brown boundary, which is defined by Eq. (18). The states with broken antisymmetry and amplitudes given by Eqs. (39) and (40) exist above the blue boundary, which is defined by Eq. (41).

In other words, for given $\varepsilon$ the symmetric state exists for *k* exceeding a value $\left( k_{\min }\right) _{{\textrm{symm}}}$ determined by Eq. (18) with < replaced by $=$, i.e., beneath the red curve in Fig. 2a. In particular,

19 $$\begin{aligned} \left( k_{\min }\right) _{{\textrm{symm}}}\approx \left\{ \begin{array}{c} 2\varepsilon ^{2},\,{\textrm{for}}\,\varepsilon \ll 1, \\ \varepsilon ^{2}/2,\,{\textrm{for}}\,\varepsilon \gg 1. \end{array} \right. \end{aligned}$$

In the SDF case, the existence area for the symmetric states is opposite, $\varepsilon >\left( \varepsilon _{\max }\right) _{{\textrm{symm}}}$. The existence boundary (18) is shown by the red curve in Fig. 2a.

The bound states (solitons) are characterized by the total power defined as per Eq. (4). For the symmetric states in the model with the SF nonlinearity, it is

20 $$\begin{aligned} P_{{\textrm{symm}}}(k)\equiv \int _{-\infty }^{+\infty }U^{2}(x)dx=\frac{1}{2} U_{{\textrm{symm}}}^{2}(k)\left[ \frac{1+\tanh \left( \sqrt{k/2}\right) }{ \sqrt{k/2}}+\frac{1}{\cosh ^{2}\left( \sqrt{k/2}\right) }\right] . \end{aligned}$$

As *k* varies from the minimum value $\left( k_{\min }\right) _{{\textrm{symm}} }$ [see Eq. (19)] towards $k\rightarrow \infty$, the power (20) grows from 0 to 2, so that

21 $$\begin{aligned} P_{{\textrm{symm}}}(k\rightarrow \infty)=2\left( 1-\varepsilon /\sqrt{2k} \right) . \end{aligned}$$

**Figure 3:**
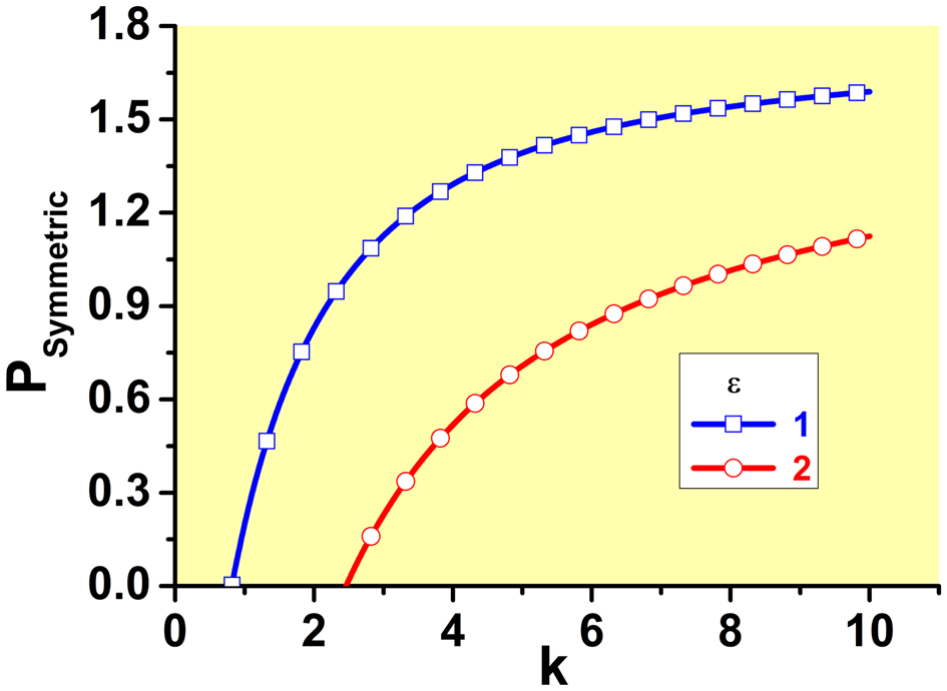
The dependence of the integral power of the symmetric bound states on the propagation constant, in the model with the SF sign of the nonlinearity, as given by Eq. (20), for $\varepsilon =1$ and 2. As shown by Eq. (21), with the increase of *k* the power is slowly approaching the limit value, $P_{ {\textrm{symm}}}(k=\infty)=2$.

Examples of this dependence for $\varepsilon =1$ and 2 are displayed in Fig. 3. Note that it satisfies the above-mentioned VK criterion, $dP/dk>0$.

An essential fact is that the substitution of ansatz (11)–(13) in Eq. (10) produces, as well, an *exact solution* for asymmetric bound states in the model with the SF nonlinearity, with the following values of amplitudes $U_{1}$ and $U_{2}$:

22 $$\begin{aligned} \left( U_{{\textrm{asy}}}\right) _{1}(k)= & {} \sqrt{\frac{E(k)+\sqrt{E^{2}(k)-4} }{2S(k)}}, \end{aligned}$$

23 $$\begin{aligned} \left( U_{{\textrm{asy}}}\right) _{2}(k)= & {} \sqrt{\frac{E(k)-\sqrt{E^{2}(k)-4} }{2S(k)}} \end{aligned}$$

(or $U_{1}\rightleftarrows U_{2}$). Typical examples of stable asymmetric states are presented in Fig. 1b. They are produced as numerical solutions of Eq. (7), being indistinguishable from their analytically found counterparts.

Obviously, the solution given by Eqs. (22) and (23) bifurcates from the symmetric one (15) (with $\sigma =+1$) at $E=2$, and exists at $E>2$. For a given propagation constant, the asymmetric solution exists if $\varepsilon$ does not exceed a respective maximum value,

24 $$\begin{aligned} \varepsilon<\left( \varepsilon _{\max }\right) _{{\textrm{asy}}}\equiv \sqrt{ 2k}\frac{1-2\exp \left( -\sqrt{2k}\right) }{1-\exp \left( -2\sqrt{2k}\right) }<\left( \varepsilon _{\max }\right) _{{\textrm{symm}}}, \end{aligned}$$

cf. Eq. (18). The boundary (24) is shown by the green curve in Fig. 2a. For fixed $\varepsilon$, the asymmetric solution exists in the region beneath this boundary, and only the symmetric state exists in the stripe between the red and green curves in Fig. 2a. In particular, $\left( \varepsilon _{\max }\right) _{{\textrm{symm}} }(k\rightarrow 0)=0$, i.e., at $\varepsilon =0$ the symmetric states exist in the entire region of $0<k<\infty$, while it follows from Eq. (24) that, in the same limit of $\varepsilon \rightarrow 0$, the asymmetric state exists, in agreement with Ref.^46^, at

25 $$\begin{aligned} k>\left( k_{\min }\right) _{{\textrm{asy}}}\left( \varepsilon =0\right) \equiv (1/2)\left( \ln 2\right) ^{2}\approx 0.24. \end{aligned}$$

In accordance with generic properties of the SSB bifurcation^23^, the symmetric states are stable solely in the stripe between the red and green curves in Fig. 2a, being destabilized by the SSB bifurcation beneath the green one. These expectations are corroborated below by direct simulations of the perturbed evolution of the symmetric modes displayed in Fig. 12.

The asymmetry degree of stationary states is defined, in terms of the respective integral power,

26 $$\begin{aligned} P\left( k\right) =\int _{-\infty }^{0}U^{2}(x)dx+\int _{0}^{+\infty }U^{2}(x)dx\equiv P_{-}+P_{+}, \end{aligned}$$

as

27 $$\begin{aligned} \Theta \equiv \frac{P_{+}-P_{-}}{P_{+}+P_{-}}. \end{aligned}$$

Full analytical expressions for the integral power of the asymmetric states, $P_{{\textrm{asy}}}(k)$, and the respective value of $\Theta$ are very cumbersome. Nevertheless, it is easy to find that, while *k* grows from the minimum value $\left( k_{\min }\right) _{{\textrm{asy}}}(\varepsilon)$ at the SSB bifurcation point, which is determined by the left inequality in Eq. (24) replaced by the equality [see, in particular, Eq. (25) for $\varepsilon =0$], towards $k\rightarrow \infty$, $P_{{\textrm{asy}}}(k)$ varies from the bifurcation-point value,

28 $$\begin{aligned} P_{{\textrm{bif}}}=P_{{\textrm{symm}}}\left( k=\left( k_{\min }\right) _{{\textrm{ asy}}}(\varepsilon)\right) , \end{aligned}$$

[with $P_{{\textrm{symm}}}(k)$ given by Eq. (20)] to

29 $$\begin{aligned} P_{{\textrm{asy}}}(k\rightarrow \infty)=1. \end{aligned}$$

Actually, Eq. (29) provides the same value as given above by Eq. (4) with $\sigma =+1$ and $k\rightarrow \infty$. It follows from the above expressions that, as $\varepsilon$ increases from zero towards infinity, $P_{{\textrm{bif}}}$ monotonously decreases from

30 $$\begin{aligned} P_{{\textrm{bif}}}\left( \varepsilon =0\right) =\frac{8}{27}\left( 3+\ln 2\right) \approx 1.094 \end{aligned}$$

to $P_{{\textrm{bif}}}\left( \varepsilon \rightarrow \infty \right) =0$. In particular, $P_{{\textrm{bif}}}\left( \varepsilon \right)$ is exponentially small for large $\varepsilon$:

31 $$\begin{aligned} P_{{\textrm{bif}}}\left( \varepsilon \right) \approx \exp \left( -\varepsilon \right) . \end{aligned}$$

Comparison of limit values (28) and (29) of the integral power for the asymmetric states makes it possible to identify a threshold value $\varepsilon _{{\textrm{thr}}}$ for the switch of the SSB phase transition between the first and second kinds (i.e., the switch between the sub- and supercritical SSB bifurcation): the phase transition may only be of the first kind for $P_{{\textrm{bif}}}>$ $P_{{\textrm{asy}}}(k\rightarrow \infty)\equiv 1$, and it becomes the second-order transition for $P_{{\textrm{bif}} }<1$. The corresponding equation, $P_{{\textrm{bif}}}=1$, combined with Eq. (24), in which $\varepsilon <\left( \varepsilon _{\max }\right) _{ {\textrm{asy}}}$ is replaced, as said above, by $\varepsilon =\left( \varepsilon _{\max }\right) _{{\textrm{asy}}}$, amounts to

32 $$\begin{aligned} 1+\tanh \left( \sqrt{k_{{\textrm{thr}}}/2}\right) +\sqrt{k_{{\textrm{thr}}}/2} {\textrm{sech}}^{2}\left( \sqrt{k_{{\textrm{thr}}}/2}\right) -2\sinh (\sqrt{2k_{ {\textrm{thr}}}})=0, \end{aligned}$$

where $k_{{\textrm{thr}}}\equiv \left( k_{\min }\right) _{{\textrm{asy}}}\left( \varepsilon =\varepsilon _{{\textrm{thr}}}\right)$. Numerical solution of Eq. (32) produces the single root, $k_{{\textrm{thr}}}\approx 0.298$, with the respective threshold value of $\varepsilon$ produced by Eq. (24):

33 $$\begin{aligned} \varepsilon _{{\textrm{thr}}}\approx 0.074. \end{aligned}$$

**Figure 4:**
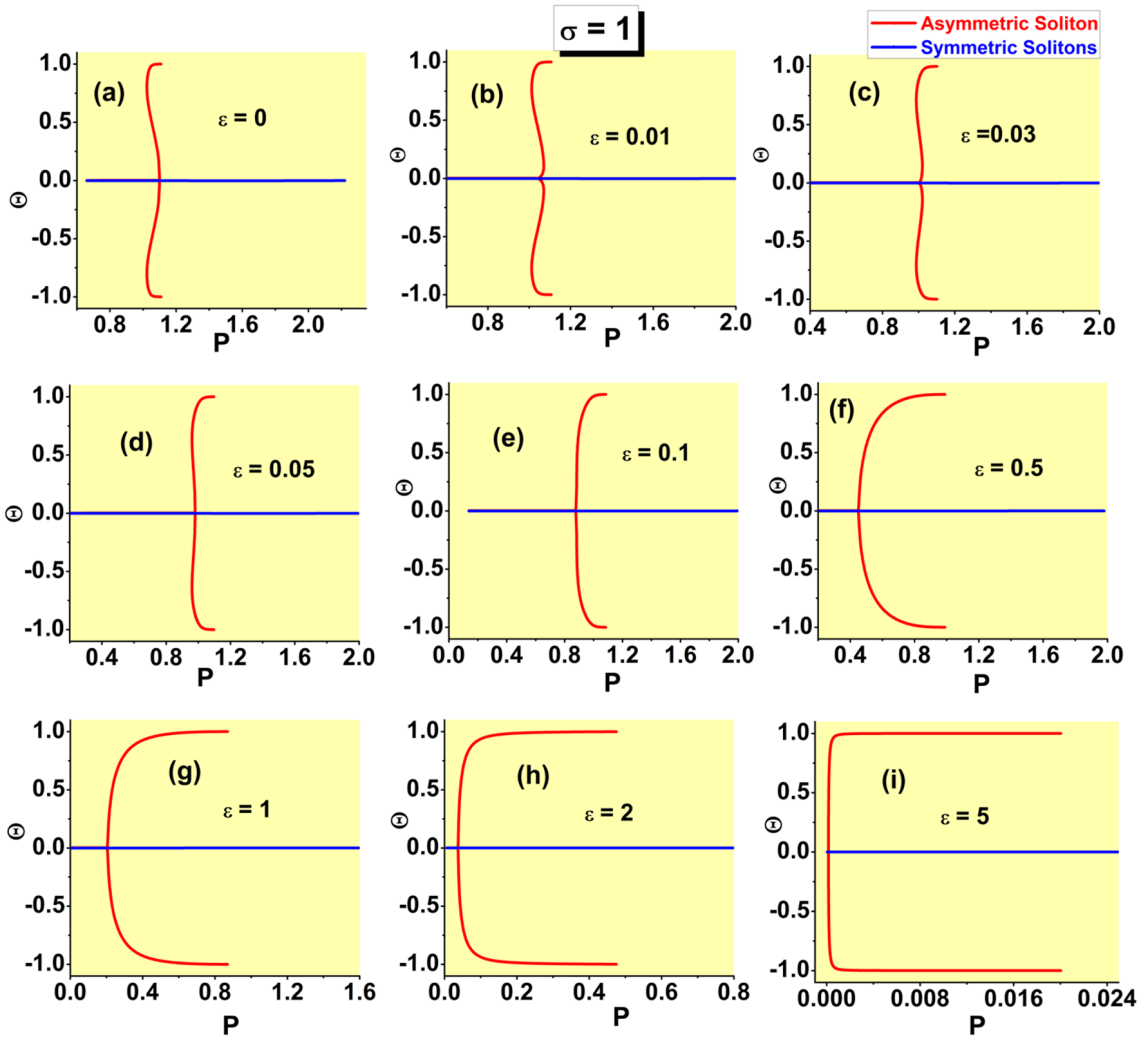
The asymmetry parameter (27) for the numerically produced solutions of Eq. (7) with the SF nonlinearity ($\sigma =+1$) vs. the integral power (26) at different values of strength $\varepsilon$ of the linear $\delta$-functional potential, which are indicated in panels. The switch between the symmetry-breaking phase transitions of the first and second kinds, alias sub- and supercritical SSB bifurcations, takes place between $\varepsilon =0.07$ and 0.08, in agreement with analytical result (33).

This result is corroborated by comparison with numerically generated SSB diagrams, in the form of $\Theta (P)$ dependences, which are displayed in Fig. 4. In a detailed form, the numerical data demonstrate that the threshold value belongs to interval $0.07<\varepsilon _{{\textrm{thr}}}<0.08$, while it is difficult to extract $\varepsilon _{{\textrm{thr}}}$ from the data with higher accuracy.

Note that narrow intervals of the variation of *P* for branches of the asymmetric states in panels (a-f) of Fig. 4 correspond to the analytical results presented here [see, e.g., the limits of the variation of *P* given by Eqs. (29) and (30)]; in panels (g-i), the $\Theta (P)$ curves are partly cut, for technical reasons. The range of the variation of *P* for the branch of the symmetric states, with $\Theta \equiv 0$, is chiefly determined by the limit value (21). For $\varepsilon =0$, the detailed analysis, reported for this case in Ref.^46^, demonstrates, in agreement with Fig. 4a, that the largest power of the symmetric solitons is $\left( P_{{\textrm{symm}}}\right) _{\max }\approx 2.08$, which is attained at $k\approx 1.40$.

The asymmetric solitons are completely stable in the area $\varepsilon _{ {\textrm{thr}}}<\varepsilon <\left( \varepsilon _{\max }\right) _{{\textrm{asy}} }$, as illustrated below by Fig. 13. At $\varepsilon <\varepsilon _{{\textrm{thr}}}$, the asymmetric solutions belonging to the lower branches in Figs. 4a–d, with $d\Theta /dP<0$, are unstable, while the upper branches, with $d\Theta /dP>0$, are stable. Actually, the instability intervals for the asymmetric solitons are very narrow.

In addition to the symmetric and asymmetric stationary states, Eqs. (6) and (6) with the SF sign of the nonlinearity, $\sigma =+1$, give rise to antisymmetric ones, with $U(-x)=-U(x)$, see an example in Fig. 1a. However, as well as in the case of $\varepsilon =0$^46^, the antisymmetric states are completely unstable because, for the same value of integral power *P*, they correspond to higher values of Hamiltonian (8) than the symmetric bound states. The instability of the antisymmetric states is illustrated below by Fig. 14.

#### The self-defocusing nonlinearity

Typical examples of antisymmetric, broken-antisymmetry, and symmetric states produced by Eq. (7) with the SDF nonlinearity, i.e., $\sigma =-1$ in Eq. (7), are displayed in Fig. 1d–f, respectively. In this case, the symmetric state, given by solution (15) with $\sigma =-1$, which exists, as said above, at $\varepsilon >\left( \varepsilon _{\max }\right) _{{\textrm{symm}}}$ [see Eq. (18)], is always stable, realizing the model’s GS. Accordingly, it is not subject to SSB. More interesting is the first excited state above the GS, i.e., the antisymmetric one, given by Eqs. (11)–(13) (with $\sigma =-1$)

34 $$\begin{aligned} U_{1}(k)=-U_{2}(k)=\sqrt{-\left[ E(k)+1\right] /S(k)}\equiv U_{{\textrm{anti}} }(k), \end{aligned}$$

where *S* and *E* is defined, as above, as per Eqs. (16) and (17). Because *S* is always positive, this solution exists under condition $E<-1$. The substitution of Eq. (17) demonstrates that this condition amounts to

35 $$\begin{aligned} \varepsilon \ge \left( \varepsilon _{\min }\right) _{{\textrm{anti}}}\equiv \frac{\sqrt{2k}}{1-\exp \left( -\sqrt{2k}\right) }, \end{aligned}$$

cf. Eq. (18). The antisymmetric state exists, at $\varepsilon >1$, in the area of the $\left( \mu ,\varepsilon \right)$ plane above the brown boundary shown in Fig. 2b. Because Eq. (35) yields $\varepsilon \ge 1$ in the limit of $k\rightarrow 0$, there are no antisymmetric states at $\varepsilon <1$. The integral power of the antisymmetric state is

36 $$\begin{aligned} P_{{\textrm{anti}}}(k)=\frac{1}{2}U_{{\textrm{anti}}}^{2}(k)\left[ \frac{1+\coth \left( \sqrt{k/2}\right) }{\sqrt{k/2}}-\frac{1}{\sinh ^{2}\left( \sqrt{k/2} \right) }\right] . \end{aligned}$$

**Figure 5:**
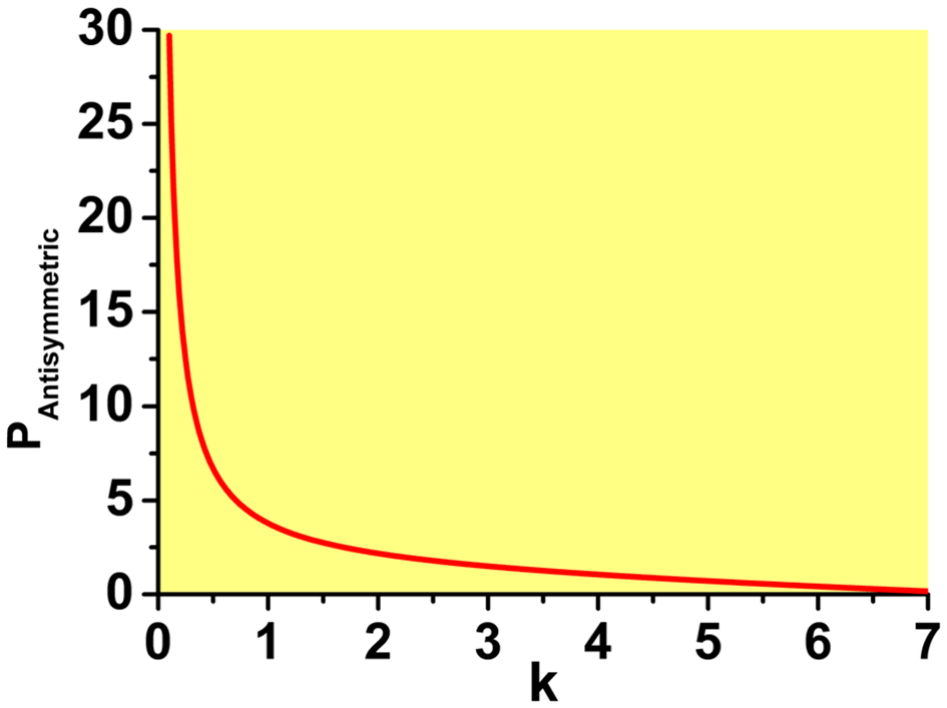
The dependence of the integral power of the antisymmetric bound states on the propagation constant *k*, in the case of the SDF sign of the nonlinearity ($\sigma =-1$), as given by Eq. (36), for $\varepsilon =4$. At $k\rightarrow 0$ the power diverges according to Eq. (37). The power vanishes at $k\approx 7.686$, which is determined by Eq. (35) with $\varepsilon =4$.

This dependence is displayed in Fig. 5 for $\varepsilon =2$. Note that expression (36) with all values of $\varepsilon$ satisfies the above-mentioned anti-VK criterion, $dP/dk<0$, which is necessary for the stability of bound states supported by the SDF nonlinearity^44^.

With the variation of *k* from the largest value, $k_{\max }$, which is determined by Eq. (35), towards $k\rightarrow 0$, the power (36) monotonously increases from $P_{{\textrm{anti}}}(k_{\max })=0$ to values diverging as

37 $$\begin{aligned} P_{{\textrm{anti}}}(k)\approx \left( \varepsilon -1\right) /\sqrt{2k} \end{aligned}$$

at $k\rightarrow 0$. The divergence is explained by the fact that, in the limit of $k\rightarrow 0$, there is an antisymmetric delocalized state with divergent power,

38 $$\begin{aligned} U_{{\textrm{anti}}}(x;k= & {} 0)=\sqrt{\varepsilon -1}{\textrm{sgn}}x,\,{\textrm{at}}\, x>+1/2\,{\textrm{or}}\,x<-1/2, \nonumber \\ U_{{\textrm{anti}}}(x;k= & {} 0)=2\sqrt{\varepsilon -1}x,\,{\textrm{at}}\,|x|<1/2. \end{aligned}$$

The bound state with broken antisymmetry is given by Eqs. (11)–(13) with amplitudes

39 $$\begin{aligned} \left( U_{{\mathrm {broken-anti}}}\right) _{1}(k)= & {} \sqrt{-\frac{E(k)-\sqrt{ E^{2}(k)-4}}{2S(k)}}, \end{aligned}$$

40 $$\begin{aligned} \left( U_{{\mathrm {broken-anti}}}\right) _{2}(k)= & {} \sqrt{-\frac{E(k)+\sqrt{ E^{2}(k)-4}}{2S(k)}}. \end{aligned}$$

This solution exists under condition $E<-2$. The substitution of expression (17) in the latter condition leads to the following existence area for the solutions with broken antisymmetry:

41 $$\begin{aligned} \varepsilon \ge \left( \varepsilon _{\min }\right) _{{\mathrm {broken-anti}} }\equiv \sqrt{2k}\frac{1+2\exp \left( -\sqrt{2k}\right) }{1-\exp \left( -2 \sqrt{2k}\right) }>\left( \varepsilon _{\min }\right) _{{\textrm{anti}}}, \end{aligned}$$

cf. Eq. (35). This area is located above the blue boundary in Fig. 2b. Because Eq. (41) yields $\varepsilon \ge 3/2$ in the limit of $k\rightarrow 0$, there are no states with broken antisymmetry at $\varepsilon <3/2$.

In agreement with the existence of the delocalized antisymmetric state (38), there is also a delocalized state with broken antisymmetry, *viz*.,

42 $$\begin{aligned} U_{{\mathrm {broken-anti}}}(x;k= & {} 0)=U_{-},\,{\textrm{at}}\,x<-1/2, \nonumber \\ U_{{\mathrm {broken-anti}}}(x;k= & {} 0)=\frac{1}{2}\left( U_{+}+U_{-}\right) +\left( U_{+}+U_{-}\right) x,\,{\textrm{at}}\,|x|<-1/2, \nonumber \\ U_{{\mathrm {broken-anti}}}(x;k= & {} 0)=U_{+},\,{\textrm{at}}\,x>-1/2, \end{aligned}$$

where

43 $$\begin{aligned} U_{\pm }=\sqrt{\frac{1}{2}\left[ \left( \varepsilon -\frac{1}{2}\right) \pm \sqrt{\left( \varepsilon -\frac{3}{2}\right) \left( \varepsilon +\frac{1}{2} \right) }\right] }. \end{aligned}$$

The mirror image of this solution is also a delocalized state with broken antisymmetry. Note that the delocalized antisymmetric state and the one with the broken antisymmetry exist, according to Eqs. (38) and (43), at $\varepsilon >1$ and $\varepsilon >3/2$, respectively, in accordance with what is said above for the generic solutions of the same types.

For the comparison’s sake, it is relevant to mention that Eq. (7) with the SF nonlinearity, $\sigma =+1$, and $\varepsilon <1$ also gives rise to a delocalized antisymmetric state with $k=0$, *viz* .,

44 $$\begin{aligned} U_{{\textrm{anti}}}^{(\sigma =+1)}(x;k= & {} 0)=\sqrt{1-\varepsilon }{\textrm{sgn}} x,\,{\textrm{at}}\,x>+1/2\,{\textrm{or}}\,x<-1/2, \nonumber \\ U_{{\textrm{anti}}}^{(\sigma =+1)}(x;k= & {} 0)=2\sqrt{1-\varepsilon }x,\,{\textrm{ at}}\,|x|<1/2, \end{aligned}$$

cf. Eq. (38). However, as well as all antisymmetric solutions of Eq. (6) with the SF nonlinearity, this solution is unstable (against modulational perturbations, cf. Rev.^38^), and Eq. (7) with $\sigma =+1$ does not produce solutions with unbroken antisymmetry.

## Numerical solutions

Numerical investigation of Eqs. (6) and (7) with the $\delta$-functions approximated as per Eq. (9) is relevant for checking the analytical results reported above. Because the width of the linear and nonlinear potential wells in real systems is finite, the numerical results also provide the verification of the relevance of the analytical predictions, which are obtained above with the use of the ideal $\delta$-functions.

### The self-focusing nonlinearity

**Figure 6:**
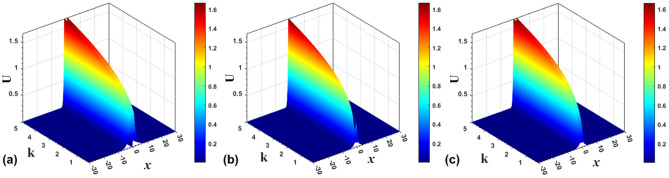
The evolution of the shapes of antisymmetric (**a**), asymmetric (**b**), and symmetric (**c**) numerically produced solutions of Eq. (7) with $\sigma =+1$ and $\varepsilon =0.5$, following the increase of *k*.

Numerically found examples of bound states of the symmetric and antisymmetric types, as well as ones with broken symmetry and antisymmetry, in the cases of the SF and SDF signs of the nonlinearity, are displayed above in Fig. 1. In a systematic way, the evolution of the antisymmetric, asymmetric, and symmetric solitons produced by Eq. (7) with $\sigma =+1$, following the increase of propagation constant *k*, is summarized in Fig. 6.

**Figure 7:**
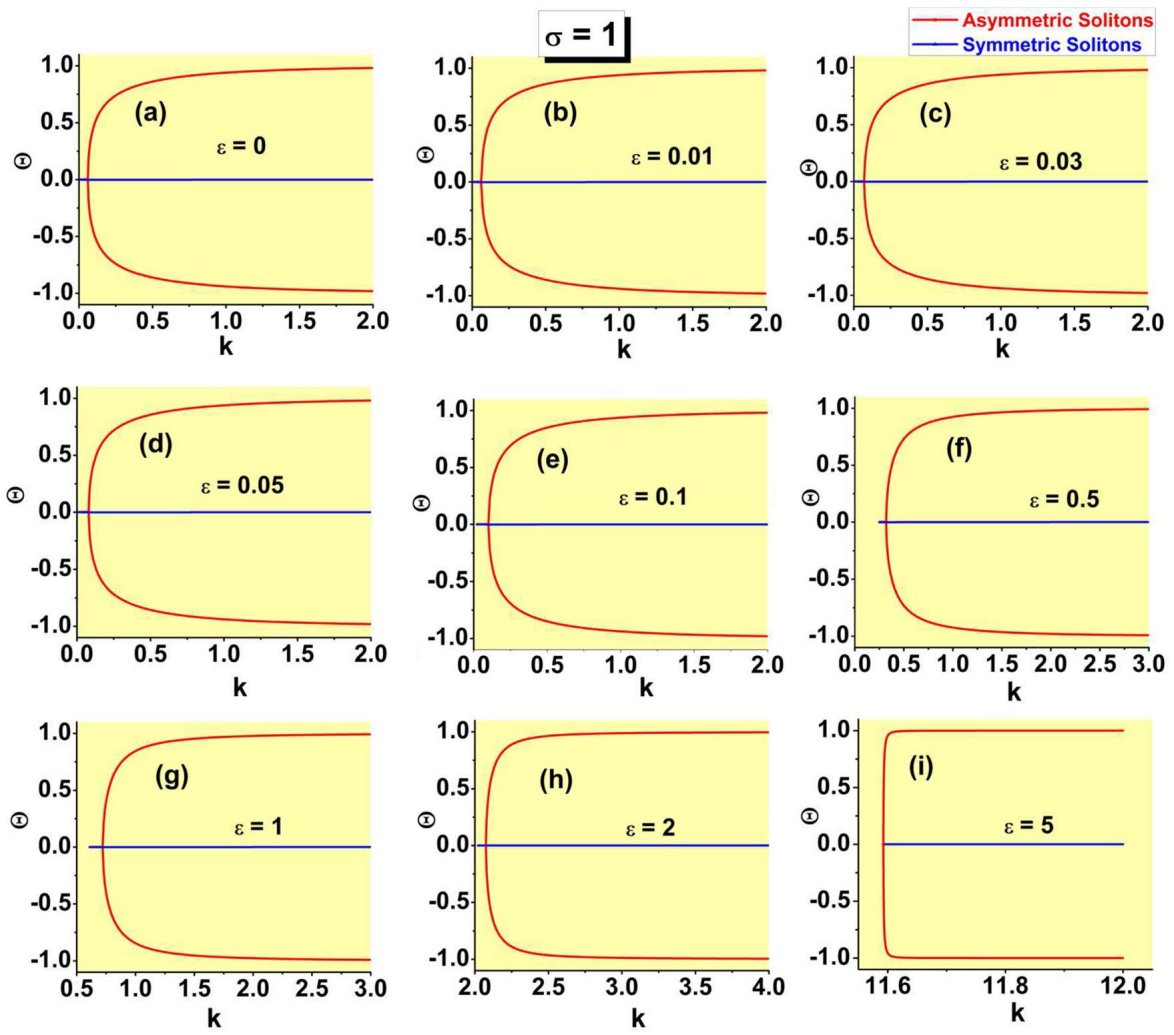
The asymmetry parameter (27) for the numerically produced solutions of Eq. (7) with $\sigma =+1$ vs. propagation constant *k* at the same values of $\varepsilon$ which are presented in Fig. 4.

The most essential results in the form of the SSB diagrams for the SF model, which corroborate the basic analytically predicted property of the model, *viz*., the switch of the character of the symmetry-breaking phase transition from the first to second kind (in other words, the switch from the subcritical SSB bifurcation to the supercritical one) at the threshold point (33), are demonstrated above in Fig. 4. In addition to that, it is relevant to plot the bifurcation diagrams in the plane of *k* and asymmetry parameter $\Theta$. These are presented in Fig. 7, for the same set of values of $\varepsilon$ as in Fig. 4. The branch of the symmetric states commences at $k=\left( k_{\min }\right) _{{\textrm{symm}}}$ [see Eq. (19)], while the value of $k(\varepsilon)$ at the SSB bifurcation point is determined by Eq. (24).

**Figure 8:**
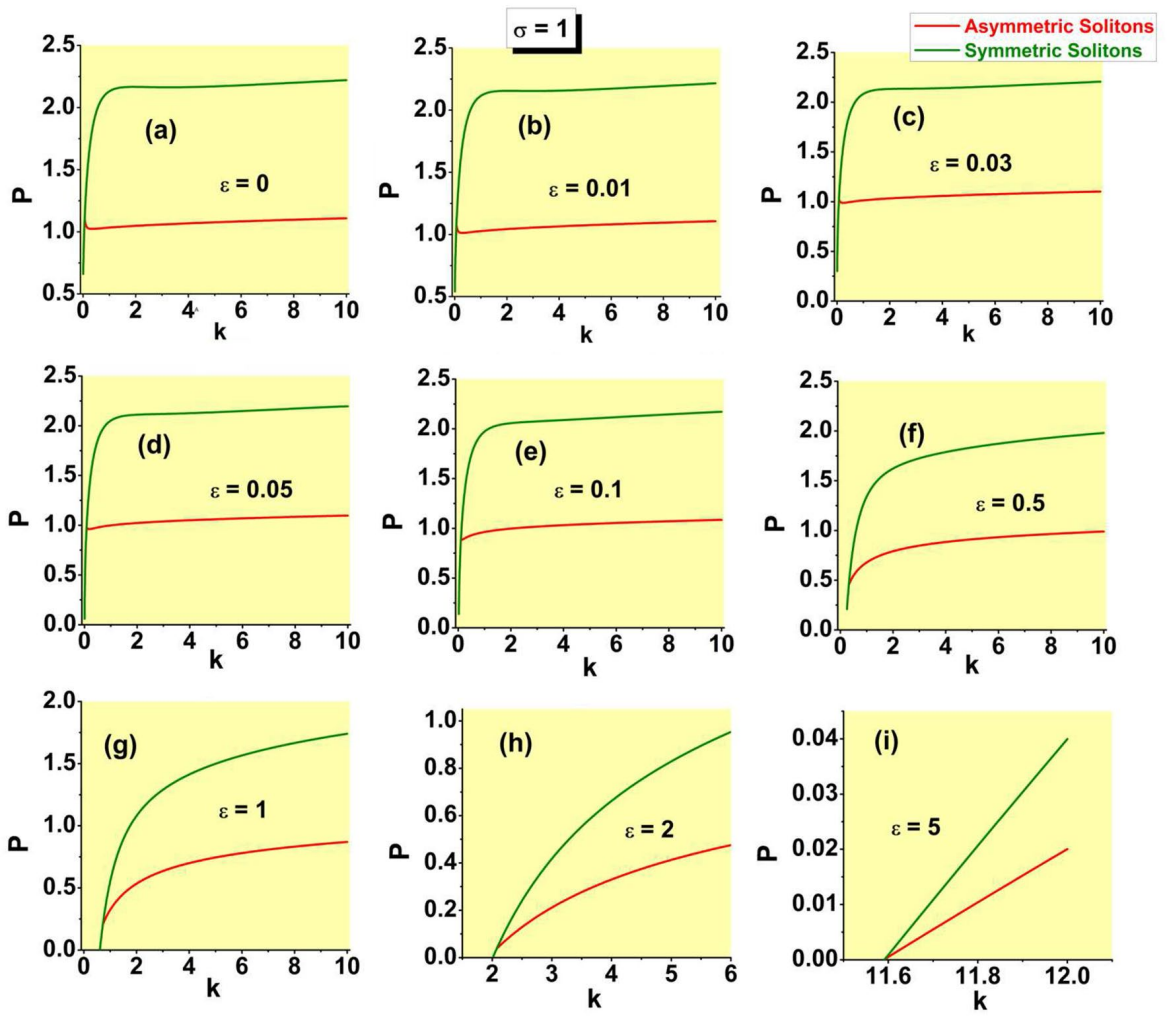
The integral power of the symmetric and asymmetric bound states in the case of the SF nonlinearity ($\sigma =+1$) vs. the propagation constant for the same values of $\varepsilon$ as in Figs. 4 and 7.

**Figure 9:**
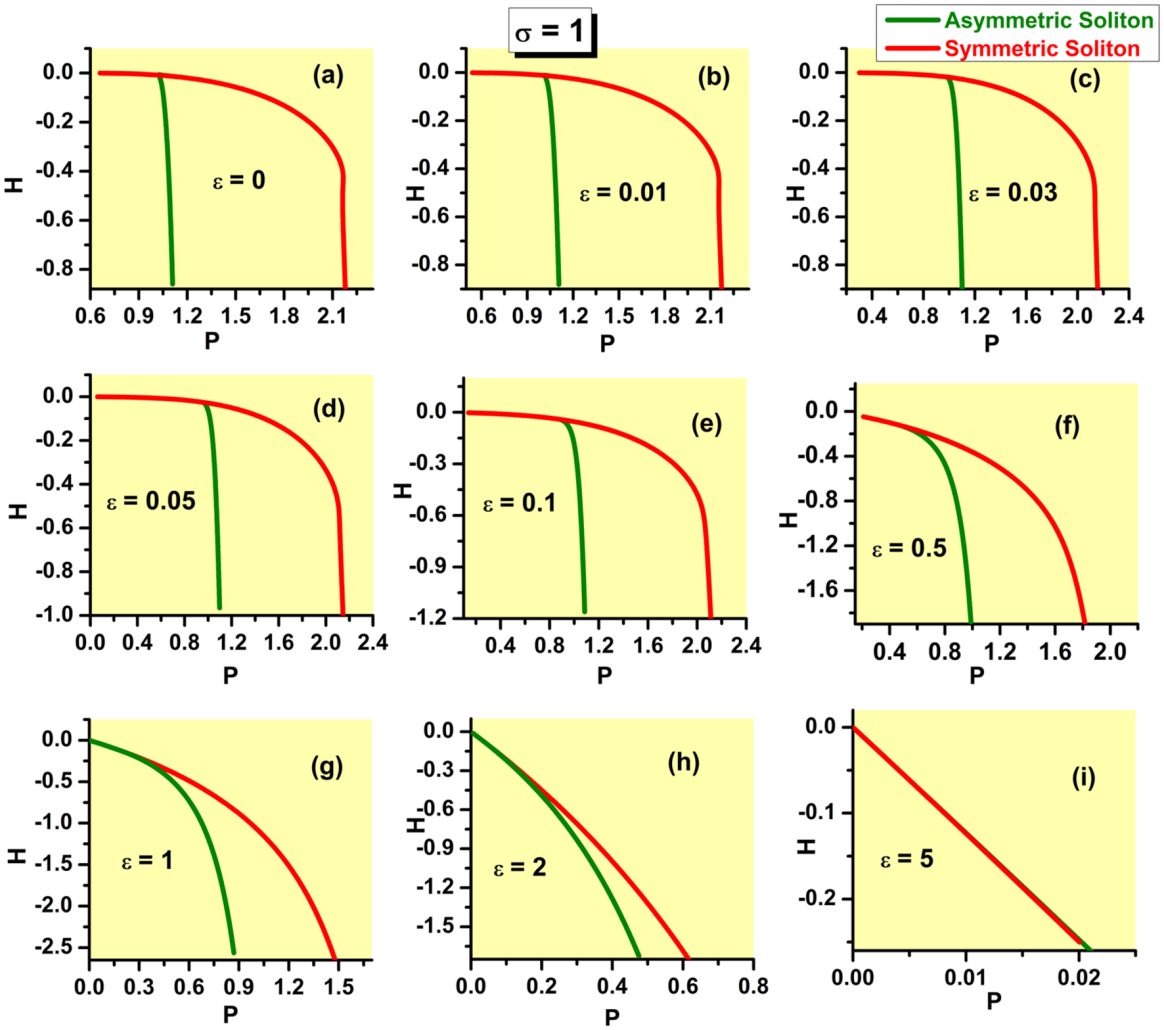
The Hamiltonian of the symmetric and asymmetric bound states, calculated as per Eq. (8) with $\sigma =+1$, vs. the integral power, *P*, for the same values of $\varepsilon$ as in Figs. 4, 7, and 8.

Further, the families of symmetric and asymmetric solitons are characterized, as physical states, by the respective dependences *P*(*k*) and *H*(*P*), where *H* is the Hamiltonian defined by Eq. (8). These dependences are displayed, respectively, in Figs. 8 and 9. In the former figure, the branches of the symmetric states commence at $k=\left( k_{\min }\right) _{{\textrm{symm}}}$, see Eq. (19). In panels (a-f) of Fig. 8, *P* varies between limit values 0 and 2 along the symmetric branches, and between $P_{{\textrm{bif}}}$ [see Eq. (28)] and $P=1$ along the the asymmetric ones [in panels (g-i), the variation range of *P* is truncated, for technical reasons; it is also partly cut in Fig. 9f]. In Fig. 9i, dependences *H*(*P*) for the symmetric and asymmetric states are indistinguishable. Note also that, in latter case, the value $P_{{\textrm{bif}}}$ at the SSB point is extremely small, in agreement with Eq. (31). Coordinates of the SSB points in Figs. 8a and 9a are correctly predicted by Eqs. (25) and (30).

In the range where the asymmetric states exist, they realizes the minimum of *H*, i.e., the system’s GS. A specific feature of the system is that it has no true GS at larger values of *P*, where only the *unstable* symmetric states exist, and there are no stationary states whatsoever at $P>2$.

### The self-defocusing nonlinearity

**Figure 10:**
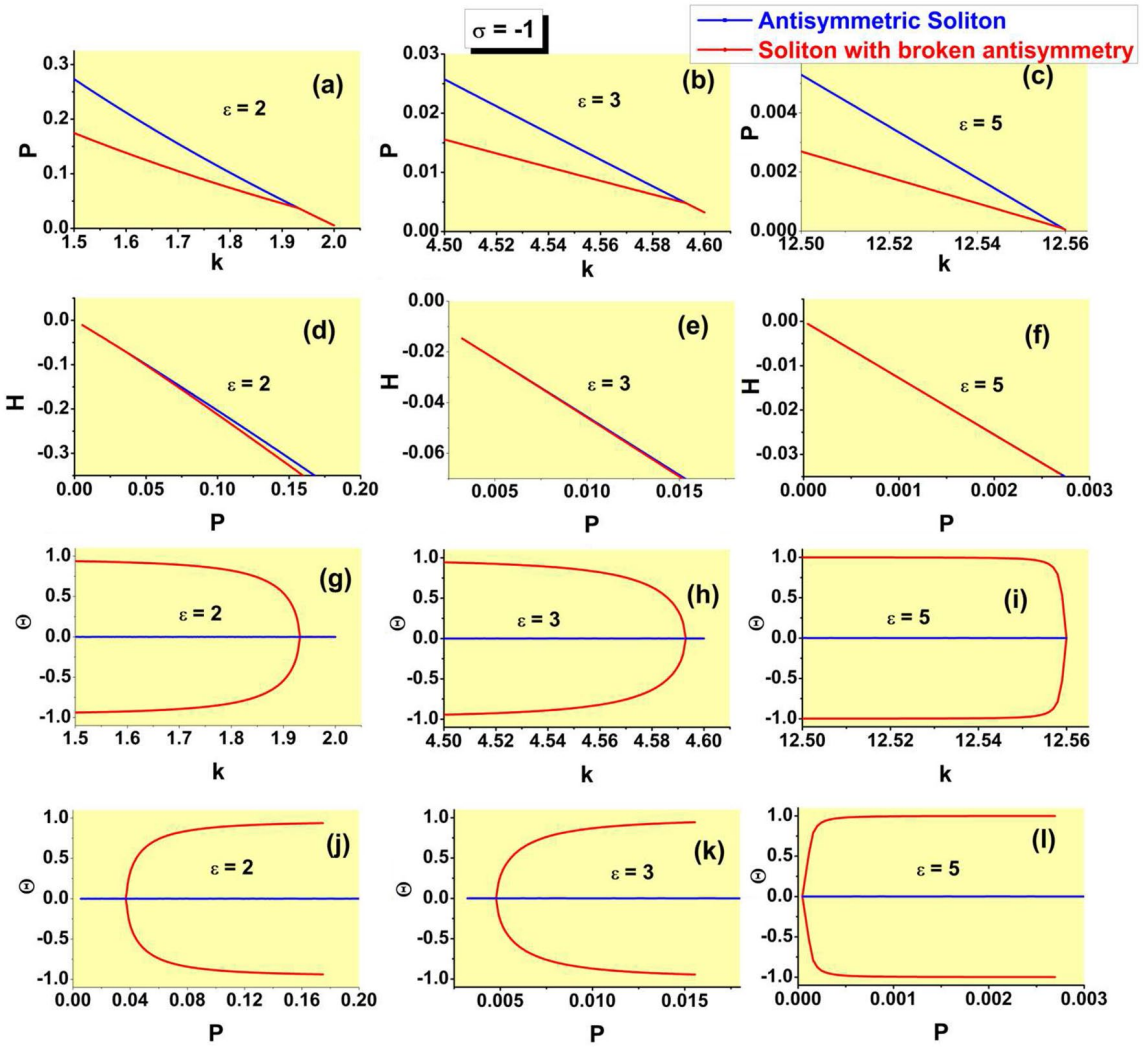
Dependences *P*(*k*) (**a**–**c**), *H*(*P*) (**d**–**f**), $\Theta (k)$ (**g**–**i**), and $\theta (P)$ (**j**–**l**) for the families of antisymmetric solitons and those with broken antisymmetry, generated by the numerical solution of Eq. ( 7) with $\sigma =-1$, three different values of $\varepsilon$, which are indicated in the panels, and the $\delta$-function approximated by expression (9). Counterparts of these dependences in the system with $\sigma =+1$ are displayed above in Figs. 8, 9, 7, and 4, respectively.

Dependences *P*(*k*), *H*(*P*), $\Theta (k)$, and $\theta (P)$ for the families of antisymmetric solitons and those with broken antisymmetry, as produced by the numerical solution of Eq. (7) with $\sigma =-1$, are collected, severally, in panels (a-c), (d-f), (g-i), and (j-l) of Fig. 10, for three different values of the strength of the linear $\delta$ -function potential, *viz*., $\varepsilon =2,3,$ and 5. These sets of plots are counterparts of those for the model with $\sigma =+1$ which are displayed above in Figs. 8, 9, 7, and 4, respectively. In particular, the *P*(*k*) curves and SSB points on all the curves plotted in Fig. 10 are correctly predicted by Eqs. (36) and (35).

An obvious difference from the case of the SF nonlinearity is that the bifurcation of the spontaneous breaking of antisymmetry in the SDF case is always supercritical, as seen in Figs. 10j–l. In other words, the model with the SDF nonlinearity always gives rise to the antisymmetry-breaking phase transition of the second kind. It is also worthy to note that the soliton branches with both unbroken and broken antisymmetry always satisfy the above-mentioned anti-VK criterion, $dP/dk<0$, which is the necessary (but not sufficient) condition for their stability.

**Figure 11:**
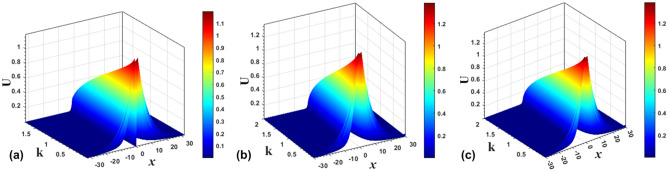
The evolution of shapes of the antisymmetric (**a**), broken-antisymmetry (**b**), and symmetric (**c**) numerically found solutions of Eq. (7) with $\sigma =-1$ and $\varepsilon =2$, following the increase of *k*.

Finally, the evolution of the antisymmetric, broken-antisymmetry, and symmetric bound states produced by Eq. (7) with $\sigma =-1$ and $\varepsilon =2$, following the increase of propagation constant *k*, is summarized in Fig. 11. Note that, in agreement with the analytical solutions, the evolution is opposite to that in the model with the SF nonlinearity ($\sigma =+1$), which is displayed above in Fig. 6. Namely, the amplitude and integral power of the solitons increase/decrease with the growth of *k* in the SF/SDF system.

### The evolution of unstable bound states

It is relevant to test the expected (in)stability of symmetric and antisymmetric bound states, as well as ones with broken symmetry and antisymmetry, in direct simulations of Eq. (6) with the ideal $\delta$-function replaced by its regularized version (9), for both the SF and SDF signs of the nonlinearity, i.e., $\sigma =+1$ and $-1$.

**Figure 12:**
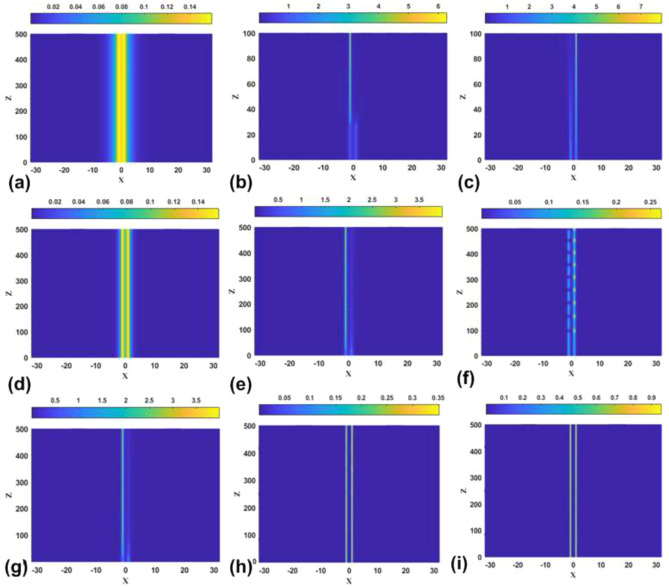
The evolution of stable and unstable symmetric bound states in the model with the SF nonlinearity, as produced by simulations of Eq. (6) with $\sigma =+1$ and parameters $(\varepsilon ,k)=(0.05,0.05)$ (**a**), (0.05, 0.1) (**b**), (0.5, 2) (**c**), (0.5, 0.3) (**d**), (0.5, 1) (**e**), (2, 2) (**f**), (2, 2.8) (**g**), (5, 10) (**h**), and (5, 11.7) (**i**). The panels plot values of $\left| \psi (x,z)\right|$ by means of the color code.

First, Fig. 12 collects typical examples which demonstrate the perturbed evolution of stable [panels (a,d,f,h,i)] and unstable [panels (b,c,e,g)] symmetric bound states in the model with the SF nonlinearity. These results are compatible with the prediction of the stability area for the symmetric states, in the form of the stripe between the lower and upper boundaries in Fig. 2a. It is observed that, naturally, all the unstable symmetric states demonstrate manifestations of the SSB instability, leading to spontaneous formation of asymmetric states. In some cases, such as the one displayed in panel 2f, the unstable symmetric state, which is located close to the instability boundary, features conspicuous persistent oscillations, while in other cases the stronger instability creates nearly stationary modes with strong asymmetry.

**Figure 13:**
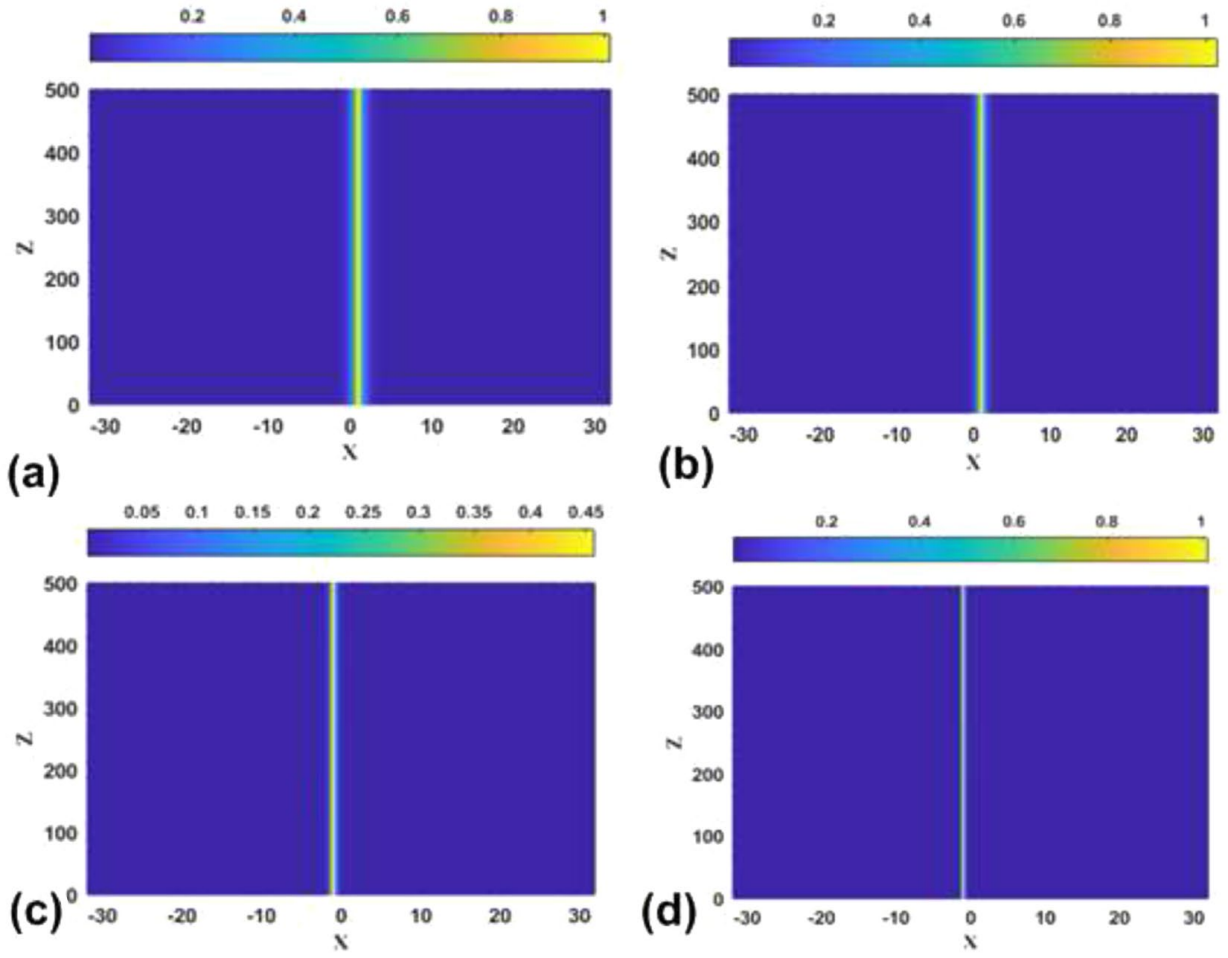
The evolution of stable asymmetric bound states in the model with the SF sign of the nonlinearity ($\sigma =+1$), as produced by simulations of Eq. (6) with $\sigma =+1$ and parameters $(\varepsilon ,k)=(0.05,0.5)$ (**a**), (0.5, 1) (**b**), (2, 2.5) (**c**), (5, 12) (**d**).

Another expected result corroborated by the direct simulations of the perturbed evolution is that nearly all the asymmetric solitons are stable in the case of the SF nonlinearity, as shown in Fig. 13 for strongly asymmetric solutions. Unstable are asymmetric solitons belonging to the backward-going (lower) branch in Figs. 4a–d. In fact, they exist only in a very narrow parameter region, and the development of the instability pulls them towards a stable counterpart existing at the same value of *P* (not shown here in detail, as this is a known feature of the subcritical SSB bifurcation).

**Figure 14:**
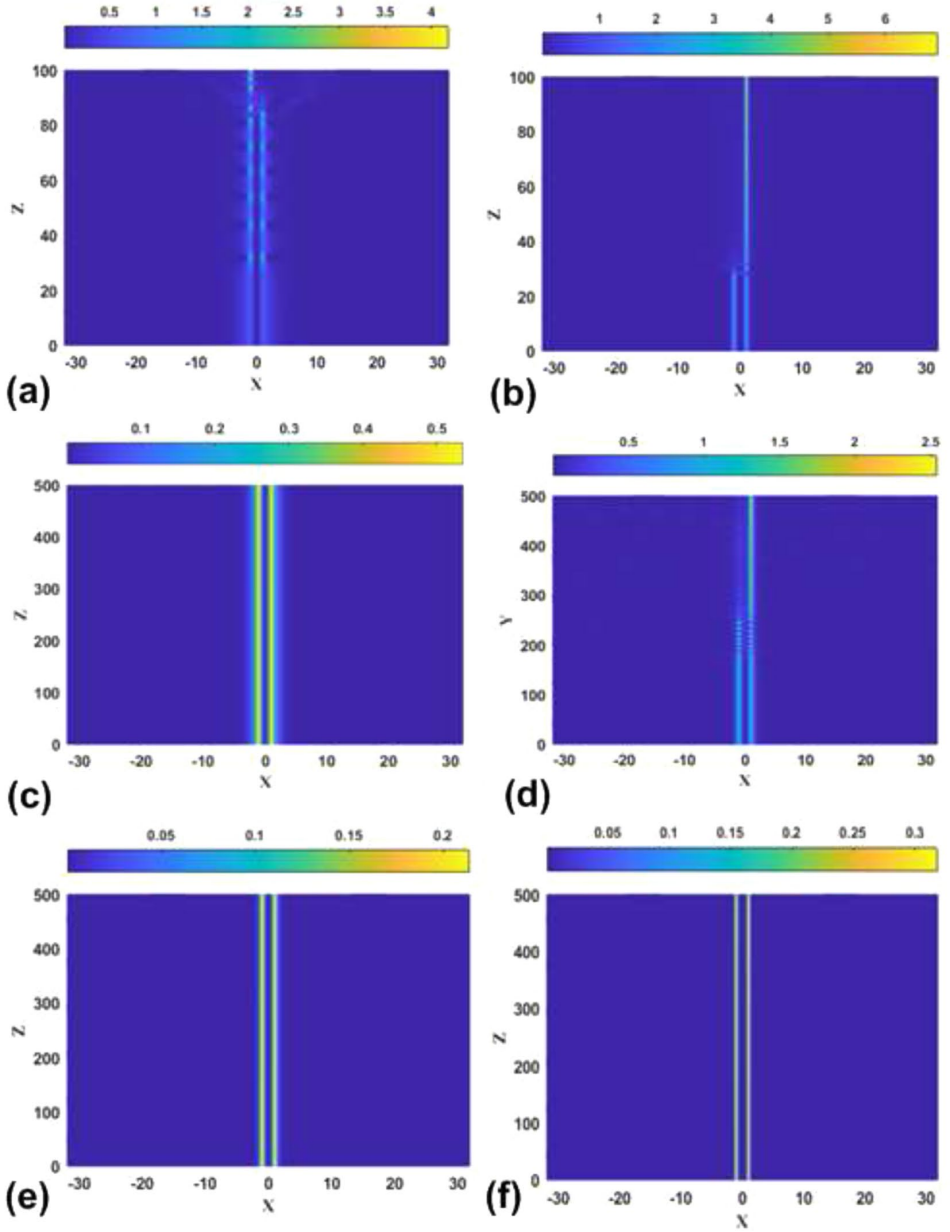
The evolution of unstable antisymmetric bound states in the model with the SF nonlinearity, as produced by simulations of Eq. (6) with $\sigma =+1$ and parameters $(\varepsilon ,k)=(0.05,0.05)$ (**a**), (0.05, 2) (**b**), (0.5, 0.3) (**c**), (0.5, 1) (**d**), (2, 2) (**e**), (5, 10) (**f**).

In addition to the above results, direct simulations displayed in Fig. 14 confirm the expected instability of all antisymmetric bound states in the case of the SF nonlinearity. In the cases shown in panels (e) and (f) of the figure, the instability is barely visible as the interaction between two power peaks of the antisymmetric modes is very weak.

**Figure 15:**
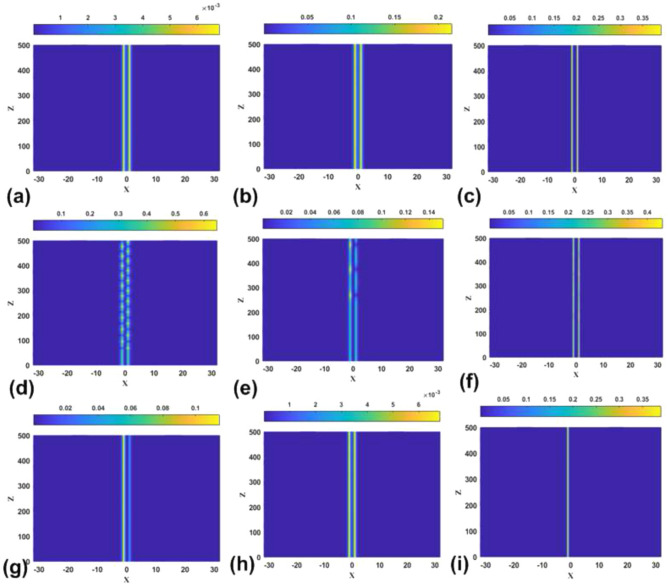
(**a**), (**b**) and (**c**): The evolution of stable symmetric bound states in the model with the SDF sign of the nonlinearity, as produced by simulations of Eq. (6) with $\sigma =-1$ and parameters $( \varepsilon ,k)=(2,1.5)$, (2, 2) and (5, 8), respectively. (**d**, **e**) and (**f**): The evolution of moderately and weakly unstable antisymmetric bound states for $(\varepsilon ,k)=(2,1)$, (2, 1.5), and (5, 8), respectively. (**g**), (**h**) and (**i**): The evolution of stable bound states with moderately, weakly, and strongly broken antisymmetry, for $(\varepsilon ,k)=(2,1.5)$, (2.8, 1.8) and (5, 8), respectively.

Lastly, characteristic examples of the perturbed evolution of the bound states of the symmetric, antisymmetric, and broken-antisymmetry types in the model with the SDF nonlinearity are collected in Fig. 15. In particular, in agreement with the above predictions all symmetric states are stable in this case, as shown in Fig. 15a–c. Further, panels (d,e) and (f) of Fig. 15 present, respectively, examples of moderately and weakly unstable antisymmetric states, in agreement with the boundaries plotted in Fig. 2b. It is seen that the instability leads to spontaneous replacement of the corresponding states by oscillatory ones with broken antisymmetry. Finally, panels (g-i) demonstrate stability of the stationary states with weakly or strongly broken antisymmetry, also in agreement with the boundary plotted in Fig. 2b.

## Discussion

Starting from the two-dimensional Ising lattice^49,50^, exactly solvable models serve as benchmarks for studies of phase transitions in diverse physical settings^51–55^. Transitions from a paramagnetic phase to a ferromagnetic one in spin systems, and similar transitions in many other media are intrinsically related to spontaneous breaking of the symmetry of the underlying setting. It is well known that phase transitions are classified as ones of the first and second kinds. Hysteresis and bistability, in the form of the coexistence of the GS (ground state) with a metastable overcooled or overheated phase, are possible in the former case.

Similar phenomenology is exhibited by nonlinear dynamical systems, in the form of bifurcations, i.e., transitions between different stable states of the system, caused by variation of the system’s control parameter(s)^23^. Counterparts of the phase transitions of the first and second kinds are identified as bifurcations of the subcritical and supercritical types in dynamical systems. The subcritical bifurcation creates stable states prior to the destabilization of the symmetric one, thus the bifurcation of this type admits the bistability and hysteresis, like phase transitions of the first kind.

In most cases, phase transitions in statistical physics, as well as bifurcations in dynamical systems, are studied between spatially uniform states. On the other hand, transitions between spatially localized (self-trapped) modes, such as solitons, are possible too. The analysis of the latter topic may benefit from the consideration of models admitting exact solutions for symmetry-breaking transitions in self-trapped states. However, finding solvable models is a challenging task, because basic integrable models that give rise to solitons, such as the one-dimensional NLSE, do not admit intrinsic transitions in the solitons.

The objective of the present work is to introduce a solvable nonlinear model with the DWP (double-well potential) which makes it possible to produce exact solutions for localized states with full and broken symmetries, that are linked by symmetry-breaking transitions of both first and second kinds. In other words, the states with unbroken and broken symmetries may be linked by bifurcations of the sub- and supercritical types. The solvability of the present model is possible due to the fact that the nonlinearity is represented by the symmetric set of two $\delta$-functions. A prototype of this model was introduced previously in Ref.^46^, but it had produced a very limited result, *viz*., the SSB (spontaneous-symmetry-breaking) bifurcation of the extreme subcritical form. That bifurcation gave rise to completely unstable asymmetric states, represented by backward-going solution branches which never turned forward. In the present work, we have introduced the solvable DWP model including both nonlinear and linear potentials, which are based on the symmetric pair of $\delta$-functions. The respective nonlinear potential is considered with both the SF (self-focusing) and SDF (self-defocusing) signs.

The analytical solutions, confirmed by their numerically found counterparts (which were produced replacing the ideal $\delta$-functions by the narrow Gaussians), give rise to the full set of symmetric and asymmetric states in the model with the SF nonlinearity, as well as the full set of symmetric and antisymmetric states, along with ones with broken antisymmetry, in the SDF model. In the case of the SF nonlinearity, the most important aspect of the analytical solution is the explicitly found switch from the symmetry-breaking phase transition of the first kind into one of the second kind, or, in other words, the switch of the subcritical bifurcation into the supercritical one. The switch takes place with the increase of strength $\varepsilon$ of the linear part of the DWP potential based on the symmetric pair of $\delta$-functions. Starting from the above-mentioned extreme subcritical bifurcation at $\varepsilon =0$, the switch is found analytically to occur at the point given by Eqs. (32) and (33), which is corroborated by the numerical findings. To the best of our knowledge, no previously studied model made it possible to predict the change of a symmetry-breaking phase transition between the first and second kinds (or the change of the sub/supercritical character of the SSB bifurcation) in an analytical form.

The analytical solution is also reported here for the model with the SDF nonlinearity, where the situation is simpler: the GS is always represented by the completely stable symmetric localized state, while the antisymmetry-breaking phase transition of the second kind (i.e., the supercritical bifurcation) destabilizes the lowest excited state (a spatially odd stationary one) at the critical point given by Eq. (41). These analytical results for the SDF model are confirmed by the numerical solution too.

The solvable models elaborated in the present work suggest possibilities for analytical studies of SSB phase transitions and bifurcations in more complex settings. In particular, it may be interesting to address a two-component system with the combined linear-nonlinear DWP potential. A degenerate form of the two-component system, with the nonlinear-only SF potential, based on the symmetric pair of $\delta$-functions, was introduced in Ref.^56^. In that model, the SF nonlinearity includes self-interaction in each component and cross-interaction between the components. Note that the two-component SF model, unlike the single-component one, admits an antisymmetry-breaking phase transition in spatially odd localized states, and it also opens the way to the consideration of the SSB transition in a state which combines spatially symmetric and antisymmetric components.

Another new possibility is offered by a model with three equidistant $\delta$-functions set on a circle, unlike the infinite one-dimensional domain considered in the present work (the circle with the purely nonlinear SDF potential, represented by a symmetric pair of $\delta$-functions set at diametrically opposite points, was addressed in Ref.^57^, in which case it did not give rise to SSB transitions, the respective GS being always symmetric). Various setups with a triangle of potential wells embedded in a nonlinear medium were studied for BEC^58,59^ and multicore optical fibers^60–64^. In the circular setting with three $\delta$-function wells, one can construct exact solutions carrying vorticity and address feasible SSB transitions in them.

On the other hand, as a step towards the consideration of two-dimensional models, where full solvability is not plausible, one can introduce a set of two parallel linearly-coupled one-dimensional lines, each bearing a DWP represented by the symmetric pair of the $\delta$-functions. In all these extensions, analytical solutions will take an essentially more cumbersome form than the one addressed in the present work, but the analysis may still be possible.

## Data Availability

The data that support the findings of this study are available from the corresponding author upon reasonable request.
